# Regulatory Testing for Endocrine Disruptors; Need for Validated Methods and Integrated Approaches

**DOI:** 10.3389/ftox.2021.821736

**Published:** 2022-01-19

**Authors:** Elise Grignard, Kelly de Jesus, Philippe Hubert

**Affiliations:** PEPPER, Paris, France

**Keywords:** endocrine disruptors, validation, regulatory testing, IATA, assays

## Introduction

The current testing of substances regarding their potential endocrine disrupting properties is hampered by a lack of validated methods. Indeed only a few endocrine pathways can be investigated by these methods, leaving many unexplored, and some adverse effects cannot be detected due to the inappropriateness of the methods, such as long term effects due to early exposure, or metabolic disruption. Moreover, *in vivo* methods are mainly based on vertebrate animals. This calls for the use of Integrated Approaches to Testing and Assessment (IATAs) for regulatory processes, which requires a comprehensive understanding of endocrine signalling mechanisms, but also an easy access to standardised methods. The relevance and reliability of new methods potentially suitable for regulatory application must be thoroughly assessed, and necessitates significant resource investment. The action of a new Public-private platform for the pre-validation of endocrine disruptors characterization methods, PEPPER, is described as well as its results.

## Current Regulatory Information Requirements Need to be Revised to Facilitate the Regulation of Endocrine Disruptors

The European Commission is Revising Information Requirements for Regulatory Testing of Endocrine Disruptors (EDs).

In September 2017 and April 2018, the Plant Protection Products and Biocide Products Regulations have respectively been amended to include scientific criteria for the determination of endocrine disrupting properties of chemicals ((EU) 2018/605 and (EU) 2017/2100).

In December 2020, out of the 22 biocidal active substances discussed by the European Chemical Agency’s (ECHA) Endocrine Disruptor Expert Group, a conclusion could be reached for only 2 (considered endocrine disruptors), while for the remaining 20, more information was needed to conclude whether the criteria were met (European Commission, 2020c). Regarding pesticides, additional data were requested by the European Food and Safety Authority (EFSA) to be able to reach a conclusion on the endocrine disrupting properties for 36% of the substances assessed for human toxicity, and for 73% of substances assessed for effects on non-target organisms (European Commission, 2020c). This inability to reach conclusions highlights the inappropriateness of the current information requirements.

In October 2020, a first step was made by the Commission towards the revision of the information requirements for identification of endocrine disrupting substances, by amending the Biocidal Products Regulation (delegated Regulation 2021/525). The amendments take into account the “need to reduce testing on vertebrate animals and the need of a testing strategy and methods for the determination of endocrine disrupting properties of substances.” Moreover, testing for endocrine disrupting properties has become part of the “core data set” (it was previously merely an “additional data set”).

Similar Activities are Ongoing Concerning Plant Protection Products and REACH Regulations.

However, including the most recently adopted OECD Test Guidelines (TGs) or their updates in the standard information requirements will not suffice to facilitate the identification of all EDs. Indeed, the available TGs mainly focus on a few endocrine pathways (i.e., estrogenic, androgenic, thyroid, as well as steroidogenesis) leaving many unexplored such as the retinoid or glucocorticoid pathways.

Another aspect to take into account when considering the revision of the information requirements is the need of methods able to fulfil the three aspects of the criteria for the identification of EDs, as laid out in the Pesticides and Biocides Regulations, i.e., the demonstration of an endocrine mode of action, an adverse effect and the biological plausible link between both. In particular the need of methods facilitating the identification of modes of action calls for the development of targeted New Approach Methods not relying on animals.

### Bridging the Gap Between Research and Regulatory Testing

Many methods exist which are relevant to the identification of EDs. However very often their development was not intended for regulatory application.

In the recent years, extensive effort has been dedicated to the identification of gaps in methods that can be readily integrated into a regulatory framework, leading to various publications (OECD, 2012; OECD, 2014b; Directorate-General for Environment European Commission et al., 2018; OECD, 2018; OECD, 2021a) and are reflected in the Fitness Check on endocrine disruptors and the Chemical Strategy for Sustainability (European Commission, 2020a; European Commission, 2020b).

The first reason for this shortage of regulatory-relevant methods is a general lack of awareness of regulatory needs from the developers of methods, so that methods may not answer the “right question.” Recently, in order to reduce the gap between research and regulatory testing, the European Commission has issued research calls asking for methods to be developed for a regulatory application [e.g., European Cluster to Improve Identification of Endocrine Disruptors (EURION) https://eurion-cluster.eu/] and intends to follow this line [European partnership for the assessment of risks from chemicals (PARC)].

Another reason is linked to the absence of documentation of the methods, pre-requisite to their transfer into a “naïve” lab and validation through a ring trial. This standardisation includes, e.g., the establishment of a Standard Operating Procedure clear enough to allow a new operator/lab to implement the method. It should describe precisely every step of the method and list technical requirements, as well as adequate acceptance criteria for the results obtained when running the method. “Historical data,” i.e., repetitions of testing of a substance using the same experimental conditions, need to be established by the developer. They will be the starting point of the demonstration of reproducibility of the method, and will facilitate the establishment of acceptance criteria for the results.

The PEPPER platform (https://ed-pepper.eu/en/) was recently (2020) created to work on this lack of standardisation. It aims to fill the gap between outputs of academic research and regulatory relevant methods, by organising pre-validations.

Doing so, PEPPER focuses on methods that target gaps identified by regulators on mechanistic or apical endpoints to improve their regulatory acceptance. For example, in its 2020 campaign, methods dealing with already reasonably covered subjects such as estrogenic and androgenic actions, or thyroid disruption were not searched for.

Identifying methods with a potential for validation and use in regulatory-relevant ED characterisation is a tricky issue for many reasons. For example, the published literature is mainly presenting toxicological properties of substances, and rarely describes methods in an extensive or transparent way. A list of data collection on methods was compiled by a group developing a case study within the context of the “Accelerating the Pace for Risk Assessment” -APCRA: AltTox, Altweb, AOP Knowledge Base, AOPwiki, BioAssay Ontology, CERAPP, CoMPARA, DB-ALM, DSSTox, eChemPortal, EU Guidance for the identification of endocrine disruptors in the context of Regulations, EPA TSCA List of Alternative Methods, OECD Conceptual Framework for Testing and Assessment of EDCs, OECD GD 150, OpenFoodTox, PubChem, QSAR Toolbox, T3DB, Tox21, ToxCast, TSAR, US EPA EDSP Test Guidelines (Dix et al., 2007; AltTox, 2010; Whetzel et al., 2011; OECD, 2013; Wishart et al., 2015; Mansouri et al., 2016; EFSA, 2018; European Chemical AgencyEuropean Food Safety Authority, 2018; OECD, 2018; Altweb, 2019; European Commission Joint Research Centre (JRC), 2019a; European Commission Joint Research Centre (JRC), 2019b; Mansouri et al., 2020; OECD, 2020; US EPA, 2020; OECD, 2021b; OECD, US EPA, 2021; US EPA, 2021b; US EPA, 2021a; US EPA, 2021c). Only 15 of these data collections actually described methods or provided links to their description. Above all, these data collections have heterogeneous contents, sizes, structures, and objectives, not to mention maintenance.

One of the main conclusions of this analysis is that there are very few data sources describing the methods in a complete enough way, such that the level of maturity of the methods (i.e; optimisation need, protocol completeness, number of tested substances) could be fully evaluated. Editors of scientific journals can require that, as supplemental materials, authors provide basic method information formatted as described in (OECD, 2014a; Krebs et al., 2019).

In addition, methods are often referred to by different names under various circumstances, which is a significant challenge. It is suggested that methods names be harmonised, and perhaps even registered and deposited into a repository created by the community.

In order to overcome the difficulties in finding test methods, a search has been conducted by PEPPER with a consortium made up of Altertox, Benaki Phytopathological Intitute, and the University of Paris. Artificial Intelligence-aided literature analysis was performed in order to identify methods with a potential to be used for regulatory purpose. Out of about 12,000 abstracts identified by the consortium, around 250 methods have been pre-selected (Zgheib et al., 2021).

Interviews of researchers, regulatory agencies, industry representatives, both about their feelings on the needs, and the analysis of expressed needs by the regulatory authorities were conducted. A side outcome of these interviews was that a lot of unpublished methods are available in research labs, which could potentially be used to answer some regulatory-relevant questions.

### PEPPER: Pre-Validation of Methods Able to Bridge Gaps

Once a list of methods of potential interest was established, a further analysis including practical considerations for a validation exercise (complexity, length of the process, availability of laboratories) reduced the number of methods to about 75. Quantification of the readiness of the method to enter pre-validation, based on a “test readiness criteria” (TRC) approach (Bal-Price et al., 2018) followed. The criteria are clustered into 13 groups, e.g., concerning the test system, the description of the exposure scheme, the availability of an SOP, the biological and toxicological relevance of the endpoints measured, or the development of a prediction model.

**FIGURE 1:**
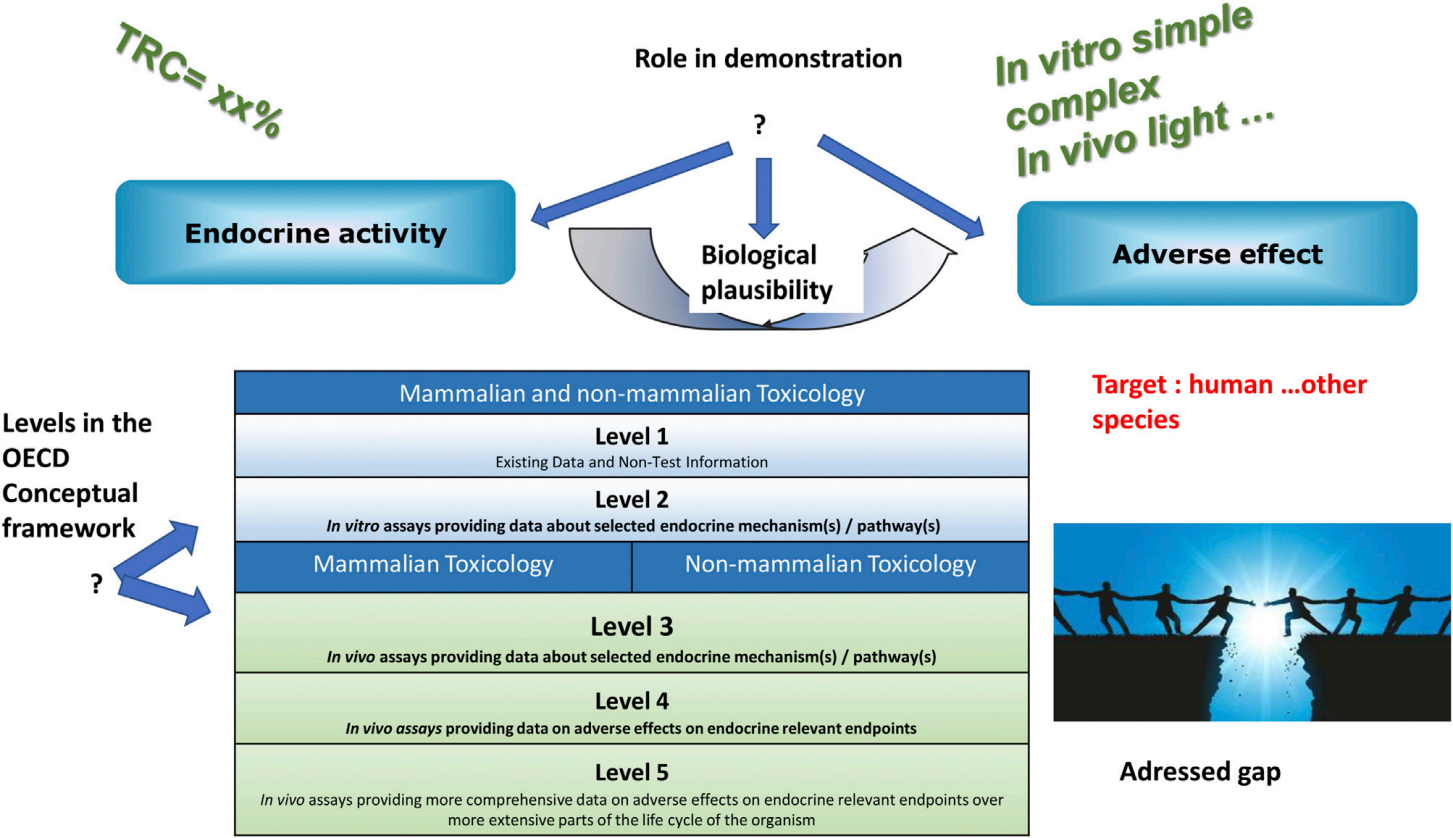
Items for the presentation of methods.

Eventually, 17 methods were considered mature enough to be presented to a Relevance Committee composed of various stakeholders, such as national and international regulatory authorities, industry, NGOs, researchers, for selection. The methods were presented along with their computed TRC, their position within the OECD Conceptual Framework for Testing and Assessment of Endocrine Disrupting Chemicals (OECD, 2018), their role in the demonstration of an endocrine disrupting property (i.e., endocrine mode of action, adverse effect, biological plausible link between both), as well as the regulatory gap addressed (Figure 1). Although it was not investigated, the link between these methods and existing AOPs would bring interesting information.

For example, the first selected method (Rat et al., 2017; Olivier et al., 2021), based on the use of human placental cells for the measurement of P2X7 activation, estradiol, progesterone, hPlacental Lactogen, and hyperglycosylated βhCG secretions, could be described as.

• having a TRC of 75% of the maximum,
• being on level 2 of the OECD Conceptual Framework,
• addressing early/intermediate Key Events
• addressing a knowledge gap on female reproduction/fertility via placental function

Another selected method bears on the glucocorticoid receptor with a transactivation approach, focusing on a very early Key Event (Chevolleau et al., 2016; Grimaldi et al., 2019).

The third ranked method, using zebrafish and looking at obesity through adipocyte size, has a 60% TRC, is on level 3 of the OECD Conceptual Framework, addresses plausibility and adverse effect (Tingaud-Sequeira et al., 2011; Elemans et al., 2019).

Once methods are chosen, field work starts with assessment of transferability, i.e., implementation of the method, following the SOP, in two or three naïve labs. This step usually leads to modifications of the SOP, e.g., to clarify some points, or make it applicable to different pieces of equipment. After successful implementation of the method, using a few test substances, the SOP is fixed and more substances are tested to further assess relevance and reliability of the method, as well as its applicability domain (OECD, 2005). The time and resource consumption needed for this work are one of the reasons why few methods are validated.

Eventually, the most tangible output will be the proposal of the methods to be included on the workplan of the OECD, to eventually be adopted as Test Guidelines. A less visible part is the dissemination of the knowledge of those methods in the European community, through participating laboratories, and scientists who help PEPPER in the validation.

## Discussion

The need for more (validated) methods in order to identify EDs is a position shared by many stakeholders, so is the recognition that (in most cases) the identification of an ED cannot rely on a single method, but requires results from several methods to be combined using a weight of evidence approach. Building an IATA can help this demonstration by providing a framework.

PEPPER is addressing the issue from a specific point of view: it identifies methods that can fill the various testing gaps, it improves the description of methods (e.g., endpoints measured, limitations, complexity) before their validation and it enhances their regulatory acceptance. The association funds and organizes the elements for validation.

It has been demonstrated that it is uneasy to find methods in the literature and in databases, that are both mature enough to enter (pre)validation and fill recognised testing gaps in ED characterisation. Moreover, the scarcity of IATAs made it almost impossible to associate the methods to IATAs.

It is not a common practice for method developers, nor in research calls, to suggest how a method could fit into a testing strategy or an IATA however it should be encouraged. Indeed, it would facilitate the regulatory uptake of the method. It seems nevertheless possible to enter a virtuous circle, with better practices in the description of methods in publications, and a greater use of databases on methods, together with an improved maintenance of the databases.
